# Enrichment of *Artemia* With Synbiotic and Its Effects on Growth Nutrient Utilization Survival and Gut Microbial Communities of Larval Hybrid Catfish (*Clarias microstomus* × *Clarias gariepinus*)

**DOI:** 10.1155/anu/6616288

**Published:** 2025-08-24

**Authors:** Arlene Debbie Lingoh, Kamil Latif, Yih Nin Lee, Lirong Yu Abit, Shahrul Razid Sarbini, Miguel Isaiah Vincent Mojilis, Fatin Maisarah Shamsul Azhar, Sabrina Rancang Khairul, Sui Sien Leong

**Affiliations:** ^1^Department of Animal Sciences and Fishery, Faculty of Agricultural and Forestry Sciences, Universiti Putra Malaysia Sarawak, Bintulu 97008, Sarawak, Malaysia; ^2^Faculty of Engineering and Sciences, Curtin University, CDT 250, Miri 98009, Sarawak, Malaysia; ^3^Department of Crop Sciences, Faculty of Agricultural and Forestry Sciences, Universiti Putra Malaysia Sarawak, Bintulu 97008, Sarawak, Malaysia; ^4^Institute of Ecosystem Science Borneo, Universiti Putra Malaysia Sarawak, Nyabau Road, Bintulu 97008, Sarawak, Malaysia; ^5^International Institute of Aquaculture and Aquatic Sciences (I-AQUAS), Universiti Putra Malaysia, Port Dickson 71050, Negeri Sembilan, Malaysia

## Abstract

*Artemia* (brine shrimp) is a vital live feed in aquaculture, providing essential nutrients during the early developmental stages of aquatic species. This study aimed to evaluate the efficacy of synbiotic-enriched *Artemia franciscana* as a live feed for hybrid catfish larvae (*Clarias microstomus* × *Clarias gariepinus*), using locally isolated probiotics (*Enterococcus faecium* and *Enterococcus faecalis*) and the commercial prebiotic inulin. The study was conducted in two phases. In Phase 1, *Artemia* were enriched for 6 h with four treatments: T1 (*E. faecium* W01 + inulin), T2 (*E. faecalis* + inulin), T3 (*E. faecium* W02 + inulin), and T4 (control and no synbiotics). Bacterial colonization was assessed microscopically and through colony counts at 2, 4, and 6 h post-enrichment. Synbiotic-treated groups (T1–T3) showed significantly higher bacterial retention than the control (T4), with T1 achieving the highest colonization levels (2 h: 6.98 log CFU/g; 4 h: 7.02 log CFU/g; 6 h: 7.10 log CFU/g; *p*  < 0.05). Control values ranged from 5.12 to 5.45 log CFU/g. Microscopy revealed a distinctive red-brown gut coloration in treated *Artemia*, indicating successful colonization. In Phase 2, hybrid catfish larvae were fed enriched *Artemia* for 7 days, followed by a subsequent 7-day period transitioned to enriched *Artemia* and commercial pellet feed. T3 resulted in the highest weight gain (263.14 ± 34.70 mg), length gain (14.38 ± 5.10 mm), specific growth rate (SGR; 19.59 ± 1.17% day^−1^), and the lowest feed conversion ratio (FCR; 0.10 ± 0.01), all significantly better than other treatments (*p*  < 0.05). Although survival rates did not differ significantly, T3 recorded the highest survival (57.5 ± 3.81%). Forty presumptive lactic acid bacteria (LAB) were isolated from the fish gut, grouped into four main clusters. These results highlight the potential of synbiotic-enriched *Artemia* to enhance larval growth and gut health, offering an eco-friendly strategy to improve feed efficiency and reduce antibiotic dependance in aquaculture.

## 1. Introduction

The rapid expansion of global aquaculture necessitates sustainable strategies to enhance fish growth, health, and survival. As one of the fastest-growing food production industries, aquaculture plays a crucial role in food security and economic development, particularly in countries like Malaysia, where freshwater species such as catfish (*Clarias* spp.) represent a significant share of national fish production [1]. However, larviculture remains a critical bottleneck due to high mortality rates, disease susceptibility, and inadequate early nutrition strategies [2]. These challenges highlight the need for innovative approaches to optimize larval nutrition and strengthen fry resilience, particularly in newly developed hybrid species like *Clarias microstomus × Clarias gariepinus*, which is the focus of this study.

Live feed organisms, especially *Artemia franciscana*, are widely used in hatchery operations due to their high digestibility, ease of culture, and ability to be enriched with essential nutrients [3]. However, conventional *Artemia* diets often lack bioactive compounds necessary for optimal larval development, potentially leading to nutrient deficiencies and increased susceptibility to environmental stressors and pathogens [4]. To overcome these limitations, this study explores synbiotic enrichment of *Artemia* as a means of enhancing live feed quality. Synbiotics, a combination of probiotics and prebiotics, have shown potential in improving gut microbiota composition, nutrient assimilation, and immune function in aquaculture species [5, 6]. While probiotics introduce beneficial microbes into the digestive system, prebiotics selectively stimulate their growth and activity, contributing to improved intestinal health and digestion [7–9]. While previous research has explored synbiotic applications in the grow-out phases of *Clarias* species, this study is among the first to investigate the early larval stage using *Artemia* as a synbiotic carrier, specifically enriched with locally isolated probiotic strains.

This study investigates a two-phase synbiotic feeding strategy in which *A. franciscana* is enriched with locally isolated probiotics (*Enterococcus faecium* and *Enterococcus faecalis*) and the commercial prebiotic inulin before being fed to hybrid catfish fry. The study aims to assess bacterial retention in *Artemia* at different time points post-enrichment and evaluate the effects of synbiotic-enriched *Artemia* on growth performance, feed conversion efficiency, and survival rates of hybrid catfish fry over a 14-day period.

The research hypothesis suggests that hybrid catfish fry fed with synbiotic-enriched *Artemia* will exhibit enhanced growth and improved survival rates compared to those fed nonenriched *Artemia*. By assessing the effectiveness of synbiotic supplementation in live feed, this study contributes to the development of sustainable aquaculture practices and optimized larval feeding strategies, ultimately supporting healthier and more efficient fish production.

## 2. Materials and Methods

### 2.1. Bacterial Strains and Prebiotics

The commercial prebiotic used in this study was inulin (Beno Orafti, Belgium). The probiotic strains used in the study were L1M10 (*E. faecalis* + inulin), L4AM2 (*E. faecium* + inulin), and L4AM5 (*E. faecium* + inulin). These strains were previously isolated from the gastrointestinal tract of village chickens and had been confirmed to possess probiotic properties [10].

### 2.2. Experimental Animal

Larvae of hybrid catfish (post hatch day-3, *n* = 480) were obtained by induced reproduction from Department of Animal Science and Fishery, Universiti Putra Malaysia Sarawak [11]. The larvae were transferred to their experimental units (housing) at the Wet Laboratory after 48 h of hatching.

### 2.3. Preparation of *Artemia* and Synbiotic Enrichment

*Artemia* (*A. franciscana*) cysts were sourced from Aquatic *Artemia*, Malaysia. The cysts underwent decapsulation using sodium hypochlorite to remove the chorionic layer. Hatching was conducted in a 120 L cone-shaped container filled with water at a salinity of 32 g/L. The decapsulated cysts were incubated at a density of 5 g/L, maintained at 30°C, under 2000 lux lighting, with continuous aeration to ensure optimal conditions. The hatched *A. franciscana* nauplii were subsequently reared in a controlled environment with a temperature range of 28–29°C, a salinity of 32 g/L, a dissolved oxygen level of 7.75 mg/L, a light intensity of 1500 lux, and a pH of 7.88. These parameters were carefully regulated to provide an optimal rearing environment.

For the synbiotic enrichment, each probiotic strain was first standardized to a concentration of 1 × 10^8^ CFU/mL using the 0.5 McFarland turbidity standard to ensure consistent cell density across treatments. Subsequently, the probiotics and inulin (5 g/L) were mixed into the culture water containing the post-hatched *Artemia* nauplii with continuous aeration to ensure homogeneous distribution of synbiotics across the culture medium. The enrichment process was conducted over 6 h to determine the optimal colonization period of the synbiotics within *Artemia*. For each time point (2, 4, and 6 h), triplicate samples of approximately 0.5 g *Artemia* (approximately 500 nauplii) were collected per treatment to assess bacterial colonization levels [12].

### 2.4. *Artemia* Gut Microflora Composition

To assess the enrichment process, samples were collected from all treatments at designated time points: 2, 4, and 6 h, following the method outlined [13]. At each time point, 100 mL of sample, containing approximately 0.5 g of *Artemia*, was collected using a sterile pipette and transferred onto filter paper. The filter paper was rinsed with a salt solution for 60 s to remove surface-associated bacteria. The washed samples were then weighed and placed in a sterile porcelain mortar for further processing.

Serial dilutions (10^−1^–10^−7^) were prepared by homogenizing the samples in a sterile saline solution (0.85% w/v). To quantify lactic acid bacteria (LAB), 0.1 mL of each dilution was spread onto de Man, Rogosa, and Sharpe (MRS) agar (Merck, Darmstadt, Germany). The plates were incubated aerobically at 30°C for 24 h. Following incubation, microbial colonies were enumerated and recorded as logarithmic colony-forming units (CFUs) per gram of *Artemia*, adjusted based on the dilution factor. The formula included are as follows:

  $$\begin{array}{c} \text{Colony forming unit (cfu/mL)}=\text{ (Number of colony}\times \text{dilution factor) / Volume of culture}. \end{array}$$

### 2.5. Microscopic Examination of Enriched *Artemia*

Gut coloration of enriched *Artemia* was evaluated using a standardized colorimetric scale (Pantone Matching System, PMS 160–175) under 400x magnification to objectively assess pigment intensity associated with synbiotic incorporation.

### 2.6. Feeding of Enriched *Artemia* to Hybrid Catfish Larvae

The experiment was conducted using a completely randomized design comprising four treatment groups. The initial phase involved a 7-day feeding trial with enriched *Artemia*, followed by a subsequent 7-day period during which larvae were transitioned to enriched *Artemia* and commercial pellet feed. The treatments included: T1 (*Artemia* enriched with *E. faecium* strain W01 and inulin), T2 (*Artemia* enriched with *E. faecalis* and inulin), T3 (*Artemia* enriched with *E. faecium* strain W02 and inulin), and T4 as the control (nonenriched *Artemia* without synbiotics). Each treatment was replicated three times, totaling 12 experimental units. The trials were conducted in rectangular plastic tanks (10 L capacity), with each tank stocked with 40 hybrid catfish larvae. The larvae had an initial average weight of 19.50 ± 0.01 mg and an average length of 4.80 ± 0.06 mm.

Fish larvae were fed *Artemia* two times daily at 12-h intervals (08:00 and 20:00). The feeding rate was adjusted to ~40–50 nauplii per larva per feeding, ensuring ad libitum intake. To ensure complete consumption, tanks were observed for 30 min post-feeding, and any uneaten *Artemia* were siphoned out and quantified. Water quality parameters, including temperature, pH, and dissolved oxygen levels, were monitored daily to ensure optimal rearing conditions. Maintaining stable water quality is essential for fish health and growth, as fluctuations can negatively impact survival and development. Any deviations from optimal conditions were promptly addressed to sustain a suitable environment for the larvae throughout the experiment.

### 2.7. Growth Performance of Hybrid Catfish Fry

Three fish was randomly collected from each tank per treatment (*n* = 36). Growth parameters of fish were measured at the beginning and end of the experiment [14]. Parameters included are as follows:

a. Weight gain (WG) = W2 (g) – W1 (mg).
b. Length gain (LG) = L2 (g) – L1 (mm).
c. Specific growth rate (SGR) = 100 × (ln (W2) - ln (W1))/*T*.
d. Feed conversion ratio (FCR) = Feed intake (mg)/Weight gain (mg).
e. Survival rate (%) = (final fish number/initial fish number) × 100.

In these formulas, W1 and W2 represented the initial and final weights, respectively, and *T* represented the number of days in the feeding period.

### 2.8. Fish Microbiota Analysis

Three fish was randomly collected from each tank per treatment. The fish (*n* = 36) were rinsed with sterile distilled water and 0.85% saline solution to remove external contaminants. For each fish, a single gut sample was collected by dissecting the entire intestinal tract to ensure comprehensive microbial representation. The extracted digestive tracts were homogenized in sterile distilled water and centrifuged to separate the supernatant. The obtained supernatant was serially diluted (10^−1^ to 10^−7^). A 1 mL aliquot of the homogenized sample was transferred into 9 mL of Man, Rogosa, and Sharpe (MRS) broth. The mixture was then pour-plated onto nutrient agar and incubated at 28°C for 24 h. Following incubation, 0.1 mL of the culture was spread onto MRS agar plates incubated at 28°C for 18–24 h under anaerobic conditions in triplicate.

### 2.9. LAB Selection

Pure bacterial isolates were selected and subjected to preliminary screening for LAB based on morphological and biochemical characteristics. *Lactobacillus plantarum* ATCC 8014 was used as control. Gram staining was performed to identify Gram-positive bacteria, followed by a catalase test using 3% hydrogen peroxide to confirm catalase-negative isolates. Nonmotility was assessed using the motility agar (Himedia, India). Additionally, glucose fermentation ability was evaluated by inoculating the isolates into MRS broth supplemented with bromocresol purple as a pH indicator. All the tests were performed in duplicate. Only isolates that were Gram-positive, catalase-negative, nonmotile, and capable of fermenting glucose were selected for further genotypic characterization.

### 2.10. Genomic DNA Extraction

Genomic DNA was extracted from Gram-positive, catalase-negative, and nonmotile bacterial isolates using the boiling centrifugation method, as described by Ng et al. [15]. A 3 mL aliquot of the incubated broth was centrifuged at 10,000 rpm for 5 min to pellet the bacterial cells. The supernatant was discarded, and the pellet was resuspended in 500 µL of sterile distilled water. The resuspended sample was then subjected to boiling at 100°C for 10 min to lyse the cells. Immediately after boiling, the suspension was cooled on ice at 4°C for 5 min. The sample was then centrifuged again at 10,000 rpm for 10 min, and the resulting supernatant was used as the DNA template for polymerase chain reaction.

### 2.11. Fish Microbiota DNA Fingerprinting

Repetitive sequence-based PCR (rep-PCR) fingerprinting was conducted using the (GTG)_5_ primer, following the protocol outlined by Leong et al. [16]. PCR amplification was performed in a thermal cycler (Bioer XP Cycler, China) using a 25 µL reaction mixture containing sterile distilled water, 5x Taq green buffer, 25 mM MgCl_2_, 25 mM deoxyribonucleotide triphosphates (dNTPs), 25 mM (GTG)_5_ primer (5′-GTGGTGGTGGTGGTG-3′), Taq DNA polymerase, and 5 µL of extracted DNA template. The PCR conditions included an initial pre-denaturation step at 95°C for 7 min, followed by four cycles of denaturation at 95°C for 2 min, annealing at 36°C for 2 min, and extension at 72°C for 2 min. This was followed by 30 additional cycles of denaturation at 95°C for 1 min, annealing at 50°C for 1 min, and elongation at 72°C for 1 min, with a final elongation step at 72°C for 5 min. The amplified PCR products were analyzed using agarose gel electrophoresis, where 5 µL of the PCR product was loaded onto a 1.5% (w/v) agarose gel stained with ethidium bromide. Electrophoresis was carried out at 90 V for 90 min, and the bands were visualized using a gel imaging system. A 1-kb DNA ladder (Promega, US) was used as a molecular weight marker to standardize the banding profiles. To determine the genetic relatedness of the bacterial isolates, a dendrogram or phylogenetic tree was constructed using the unweighted pair group method with arithmetic mean (UPGMA) and Dice's coefficient for cluster analysis.

### 2.12. Statistical Analysis

The data collected were subjected to statistical analysis using the statistical analysis system (SAS) software version 9.4. analysis of variance (ANOVA) was conducted to determine the significance of differences among the treatments, followed by Duncan's multiple range test for post-hoc comparisons. Significance was set at *p*  < 0.05, and the results were presented as mean ± standard deviation.

## 3. Results and Discussions

### 3.1. Microscopic Visualization

**Figure 1:**
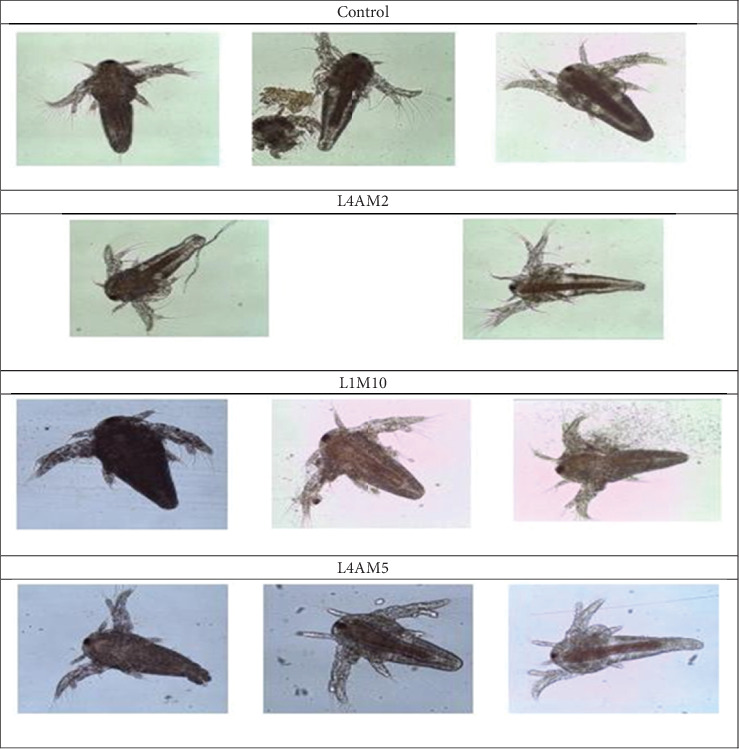
Microscopic visualization (400x magnification) of *A. franciscana* gut following exposure after 6 h postsynbiotic enrichment treatments. Treaments: T1: L4AM2 (*E. faecium* W01 + inulin); T2: L1M10 (*E. faecalis* + inulin); T3: L4AM5 (*E. faecium W02* + inulin); T4: control (no synbiotics).

Figure 1 presents the microscopic visualization (400x magnification) of *A. franciscana* gut following exposure to different enrichment treatments.

Microscopic examination at 400x magnification of *Artemia* guts post-enrichment revealed a significantly higher presence of live bacteria in the synbiotic-treated groups (T1, T2, and T3) compared to the control (T4). The enriched *Artemia* exhibited a distinct gut coloration ranging from light brown to reddish-brown (Pantone 161–174), indicative of synbiotic incorporation, as similarly observed in probiotic-enriched live feeds shown in Chittapun et al. [17]. Compared to the control, synbiotic-treated *Artemia* showed a visibly expanded abdominal region under microscopy, likely reflecting increased microbial colonization and possible enhancement of nutrient assimilation capacity. Recent research has highlighted *Artemia* as an effective bio-capsule for delivering beneficial microbes in aquaculture systems. Nikapitiya et al. [18] demonstrated that *Artemia* enriched with bacteriophages could serve as a viable vehicle for transferring these microbes into cultured fish, thereby, promoting fish health. Several studies have explored the application of probiotics in *Artemia* and other live feeds to enhance their nutritional value and improve aquaculture outcomes. For instance, *Artemia nauplii* have been successfully enriched with *Lactobacillus sporogenes* and fed to freshwater prawn (*Macrobrachium rosenbergii*) post-larvae, resulting in improved growth performance, survival rates, and biochemical composition [19]. Similarly, bioencapsulation of probiotic *Bacillus* species in *Artemia* has been shown to effectively deliver beneficial microbes to the digestive tracts of fish larvae [20, 21] and prawn [21], supporting gut health and enhancing larval development [22]. Beyond *Artemia*, probiotic enrichment of other live feeds such as rotifers and copepods has also been investigated as a strategy to improve the nutritional quality and immune-boosting properties of these organisms, further benefiting fish larvae during early development [23]. These findings highlight the potential of probiotic-enriched live feeds as an effective tool for promoting growth, survival, and disease resistance in aquaculture systems.

### 3.2. Bacterial Counts

**Figure 2:**
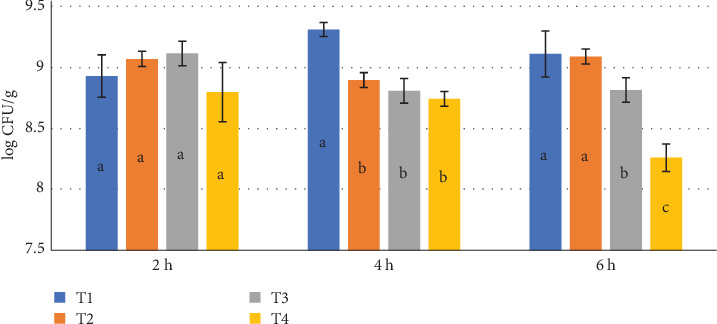
Colony-forming unit (log CFU/g) counts of lactic acid bacteria in *Artemia* sampled at 2, 4, and 6 h postsynbiotic enrichment, including T1 (*E. faecium W01* + Inulin), T2 (*E. faecalis* + Inulin), T3 (*E. faecium W02* + Inulin), and T4 (control, no synbiotics). abcd: Same letters indicate no significant difference between the groups (*p*  > 0.05).

Figure 2 illustrates the CFU counts of LAB in *Artemia* sampled at 2, 4, and 6 h postsynbiotic enrichment. The bacterial counts exhibited significant variations among treatments and time points, highlighting the effectiveness of the enrichment process. At the 2-h mark, Treatment T1 (*E. faecium* + inulin) recorded the highest bacterial load, reaching an average of 6.98 log CFU/g. Treatments T2 (*E. faecalis* + inulin) and T3 (*E. faecium* + inulin) followed closely with bacterial counts of 6.75 log CFU/g and 6.65 log CFU/g, respectively. The control group (T4) exhibited the lowest bacterial count at 5.12 log CFU/g. The rapid increase in bacterial load among the synbiotic treatments suggests efficient colonization of probiotics within *Artemia*.

At the 4-h time point, all synbiotic treatments maintained high bacterial counts, with T1, T2, and T3 registering 7.02 log CFU/g, 6.90 log CFU/g, and 6.80 log CFU/g, respectively. Meanwhile, the control group exhibited a slight increase to 5.30 log CFU/g, though it remained significantly lower than the synbiotic-enriched groups. These findings suggest that *Artemia* enriched with synbiotics can effectively retain high probiotic loads over an extended period, ensuring a sustained beneficial microbial presence. The result was concurrent with Azimirad et al. [13] demonstrated that nauplii *Artemia* can retain a large amount of probiotic by extended period. By the 6-h sampling time, T1 continued to exhibit the highest bacterial counts at 7.10 log CFU/g, followed by T2 and T3 with 6.95 log CFU/g and 6.85 log CFU/g, respectively. The control group showed a marginal increase to 5.45 log CFU/g. The consistently high bacterial retention in the synbiotic treatments indicates that enriched *Artemia* can serve as an effective bio-carrier of probiotics, potentially enhancing their nutritional and functional value as live feed in aquaculture.

Statistical analysis using ANOVA and Duncan's multiple range test confirmed significant differences (*p*  < 0.05) in bacterial counts among treatments and across time points. The synbiotic-enriched groups (T1, T2, and T3) consistently exhibited significantly higher bacterial counts compared to the control (T4) at all time intervals, reinforcing the efficacy of synbiotic enrichment in enhancing microbial colonization within *Artemia*.

The notable increase in bacterial counts observed in synbiotic-enriched *Artemia* indicates an enhancement in their nutritional profile. *Enterococcus faecium* and *E. faecalis*, as probiotic strains, are widely recognized for their positive effects on host organisms, including improved digestive efficiency, strengthened immune response, and increased resistance to pathogenic infections [24]. The inclusion of inulin as a prebiotic further facilitates the proliferation and activity of these probiotics, fostering a synergistic interaction that amplifies their overall health benefits [25]. These findings are consistent with previous studies demonstrating that synbiotic supplementation can address nutritional deficiencies in live feed, ultimately improving the health, growth performance, and survival of fish larvae [26–28].

### 3.3. Growth Performance and Survival of the Larvae

The findings presented in Table 1 highlighted the substantial impact of synbiotic-enriched *Artemia* on the growth performance and survival of hybrid catfish larvae over the 14-day experimental period. Fry-fed with synbiotic-enriched *Artemia* exhibited significantly superior growth parameters compared to the control group (T4). Among the treatments, T3 (*E. faecium* + inulin) demonstrated the highest weight gain (263.13 ± 34.70 mg) and length gain (14.38 ± 5.10 mm), both of which were significantly greater than the other treatments (*p*  < 0.05). Similarly, the SGR was highest in T3 (19.53 ± 1.17% day^−1^), indicating enhanced growth efficiency. The high variation in SGR despite relatively small differences in length gain may be attributed to the circular or bulk-like growth pattern typical of hybrid *Clarias* species, where mass increases disproportionately compared to linear body extension. This growth trend has been observed in larval and juvenile *Clarias* hybrids, where somatic tissue develops more volumetrically than longitudinally under optimal nutritional conditions [29]. The FCR for all four treatments ranged 0.10 ± 0.01 to 0.59 ± 0.10, which is very low compared to ideal FCR ratio.

FCR was estimated based on the number of *Artemia* administered and the corresponding biomass gain recorded over the 7-day feeding period. The observed low FCR values may be attributed to the high digestibility and nutrient availability of live *Artemia*, which are known to enhance feed utilization efficiency in early larval stages [30]. Tanks were cleaned regularly to minimize the accumulation of biofilm or detritus, and no supplemental feed or algal growth was observed during the trial. Nonetheless, we recognize the potential contribution of residual organic matter or microbial film to larval nutrient intake, which may have influenced FCR outcomes. Future studies should incorporate more precise quantification of total ingested biomass and environmental nutrient sources to better evaluate feeding efficiency. This finding underscores the role of synbiotics in improving nutrient absorption and metabolic efficiency, as probiotics contribute to digestive enzyme activity [31] while prebiotics create a favorable environment for beneficial gut microbiota [32]. The improved FCR observed in this study aligns with previous research, which has demonstrated that synbiotic supplementation enhances feed efficiency by promoting gut microbial equilibrium and optimizing intestinal morphology in fish larvae [8]. The high variance in growth rate among treatments may reflect individual differences in feed intake and assimilation efficiency, which are common in larval stages of hybrid *Clarias* due to uneven competition and rapid metabolic shifts. Feed intake was indirectly assessed by monitoring daily *Artemia* consumption per tank, adjusted for biomass, and ensuring no visible feed remained after each feeding session to maintain consistent intake across replicates.

Although survival rates did not differ significantly among treatments (*p*  > 0.05), T3 exhibited the highest survival rate (57.5 ± 3.81%), suggesting a potential protective effect of synbiotic supplementation. The overall survival rate was relatively low, likely due to the restricted *Artemia* feeding period of 7 days combined with a twice-daily feeding frequency. These limitations may have contributed to the high mortality observed and the absence of significant differences in survival among treatments. This is acknowledged as a key constraint in the current study design. Previous studies have reported that synbiotics enhance disease resistance and stress tolerance in aquaculture species by modulating gut microbiota and strengthening immune responses [33]. The modest improvement in survival rates observed in this study may be attributed to the immunostimulatory properties of *Enterococcus* spp., which have been shown to enhance mucosal immunity and increase resistance to pathogens in fish [34].

The enhanced growth performance observed in the T3 group suggests that synbiotic-enriched *Artemia* improves nutrient bioavailability, digestion efficiency, and metabolic function in hybrid catfish fry. Similar findings were reported by Say et al. [35], who demonstrated that hybrid catfish (*C. gariepinus × C. macrocephalus*) fed a synbiotic mixture of chitosan and Acinetobacter KU011TH exhibited increased goblet cell counts in the midgut, indicating enhanced nutrient absorption and immune function. Although the specific probiotic strains differ, both studies highlight the role of synbiotics in improving gut morphology and microbial balance, leading to better digestion and nutrient assimilation.

*E. faecium* has been widely recognized for its positive effects on gut health, particularly due to its ability to produce antimicrobial peptides, strengthen gut epithelial integrity, and competitively exclude pathogens. One key mechanism underlying these benefits is the production of bacteriocins and antimicrobial peptides that inhibit pathogenic bacteria, thereby, promoting a healthier gut microbiota [36]. By suppressing harmful microbes and fostering beneficial bacteria, *E. faecium* contributes to enhanced digestion, immune function, and overall fish health. Inulin, as a prebiotic, provides fermentable carbohydrates that selectively stimulate beneficial bacteria, reinforcing the synergistic effects of probiotics. Its fermentation by gut microbiota supports the proliferation of beneficial species, thereby, improving digestive efficiency and overall health. This effect has been observed in aquaculture species, as demonstrated by Defaix et al. [37], who reported that dietary inulin supplementation in rainbow trout (*Oncorhynchus mykiss*) enhanced growth performance and exhibited anti-inflammatory properties.

The improved microbial quality of synbiotic-enriched *Artemia* is expected to positively influence the growth and survival of fish larvae. Enrichment of live feed, such as *Artemia*, plays a critical role in providing essential nutrients for larval development, survival, and immune competence [38]. Probiotics, particularly *Enterococcus* spp., have been shown to enhance nutrient absorption, boost immune responses, and offer protection against pathogens [32]. The present study indicates that synbiotic-enriched *Artemia* serves as a more nutritionally balanced diet for fish larvae, potentially leading to higher survival rates and improved growth performance. Similar results have been reported in other species, as shown by Elshafey et al. [39], who found that feeding goldfish (*Carassius auratus*) with enriched *Artemia* improved growth, health status, and immune-physiological responses. These findings suggest that the advantages of synbiotic enrichment extend across various fish species, reinforcing its potential as a valuable strategy in larval nutrition.

### 3.4. Fish Microbiota Analysis

**Figure 3:**
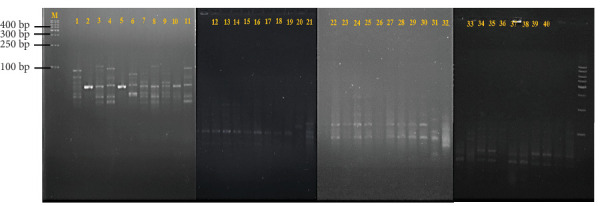
Banding profiles of GTG_5_ PCR for LAB isolated from the gut of the hybrid catfish. Lane M: 1 kb DNA ladder (Promega, USA), NC: negative control, 1: T1 (1); 2: T1 (2); 3: T1 (3); 4: T1 (4); 5: T1 (5); 6: T1 (6); 7: T1 (7); 8: T1 (8); 9: T1 (9); 10: T1 (10); 11: T2 (1); 12: T2 (2); 13: T2 (3); 14: T2 (4); 15: T2 (5); 16: T2 (6); 17: T2 (7); 18: T2 (8); 19: T2 (9); 20: T2 (10); 21: T3 (1); 22: T3 (2); 23: T3 (3); 24: T3 (4); 25: T3 (5); 26: T3 (6); 27: T3 (7); 28: T3 (8); 29: T3 (9); 30: T3 (10); 31: T4 (1); 32: T4 (2); 33: T4 (3); 34: T4 (4); 35: T4 (5); 36: T4 (6); 37: T4 (7); 38: T4 (8); 39: T4 (9); 40: T3 (10).

**Figure 4:**
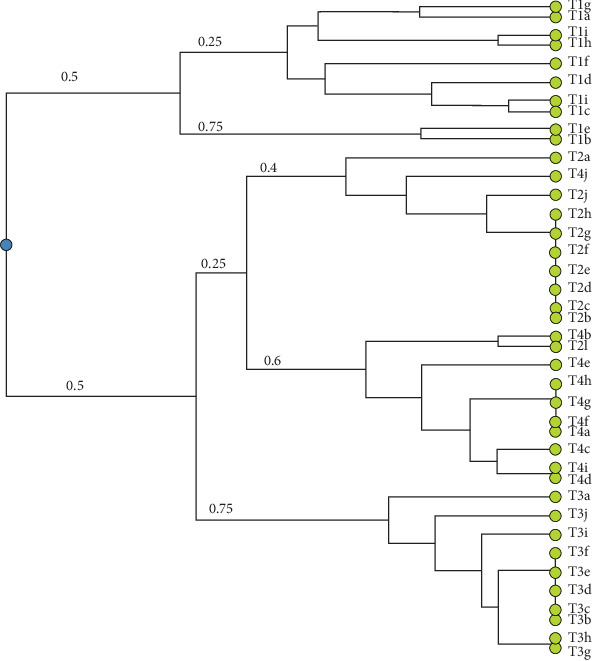
Dendogram clustering of the lactic acid bacteria isolates from the gut of hybrid catfish after 14 days feed with synbiotic enriched *Artemia*.

A total of 40 LAB isolates were successfully obtained from the gut of hybrid catfish. A higher abundance of presumptive LAB were recovered from fish in Treatment T1–T3, which showed the best probiotic effect, compared to the control group (T4), where fewer and less diverse isolates were detected. The banding profiles of these isolates, analyzed through genotypic (GTG)_5_ fingerprinting, are presented in Figure 3. Cluster analysis using a dendrogram (Figure 4) revealed four distinct main clusters, indicating genetic diversity among the isolates.

The gastrointestinal tract of fish is a vital organ system responsible for digestion and nutrient absorption, directly influencing growth performance [40]. Moreover, it serves as a complex ecosystem housing a dense and diverse microbial community, with microbial composition varying across different sections of the gut. This study found that bacterial distribution differed across intestinal sections, with bacterial populations being most abundant in the large intestine when cultured under acidic conditions. Phenotypic characterization successfully identified 40 presumptive Gram-positive LAB isolates, reinforcing the presence of beneficial *Lactobacillus* strains within the fish gut microbiota.

The findings of this study indicate that LAB populations were significantly higher in the gut of treated fish compared to the control group. Furthermore, the gut microflora in the treated fish exhibited greater diversity, with an increased abundance of beneficial *Lactobacillus* species. This aligns with the study by Elidrissi et al. [41], who investigated *Lactobacillus* strains isolated from fish intestines and reported that these strains demonstrated potent inhibitory effects against pathogenic fish bacteria. This suggests that the presence of LAB in the fish gut contributes to pathogen suppression, thereby promoting gut health and reducing the risk of infections. LAB are widely recognized as probiotics due to their prevalence in fermented foods and their classification as Generally Recognized as Safe (GRAS) microorganisms. Probiotic LAB strains play a crucial role in maintaining gut homeostasis by inhibiting pathogenic bacteria, enhancing nutrient absorption, and improving overall host health. However, bacterial identification remains a challenge due to genetic and phenotypic variations. According to Yee et al. [42], discrepancies often arise between genotypic and phenotypic characterization methods for LAB and other bacteria. Our study can classify the LAB bacteria into four groups (Figure 4). Molecular tools become essential for subspecies-level identification. Similarly, Leong et al. [16] reported that discrepancies in biochemical test results could be attributed to plasmid loss during bacterial culture, further complicating accurate classification, thus molecular identification are more reliable.

Our study investigates the probiotic effects of LAB in *Artemia* and hybrid catfish. Probiotic bacteria must exhibit resilience to harsh gastrointestinal conditions, such as acidic environments and high bile salt concentrations, to effectively colonize the gut. According to Latif et al. [43], probiotics must tolerate low pH conditions and efficiently utilize glucose as an energy source to sustain their metabolic activities. In this study, *Artemia* enriched with probiotics may serve as a vector for delivering beneficial bacteria to the gut of hybrid catfish, potentially improving their health and growth. A total of 40 LAB isolates were successfully obtained from the gut of healthy hybrid catfish, demonstrating key probiotic properties, including acid tolerance and the ability to survive under adverse gastrointestinal conditions. Notably, LAB strains capable of withstanding bile salt exposure also exhibited efficient bile salt hydrolysis, highlighting their adaptability and survivability within the digestive system [10, 44]. Furthermore, our results indicated that dietary supplementation with LAB significantly enhanced the growth performance of hybrid catfish. Fish in the T3 treatment group exhibited a notable increase in weight gain and SGR, suggesting that LAB supplementation positively influenced feed conversion efficiency and overall fish development. These findings underscore the potential application of LAB as a probiotic in aquaculture to improve fish health and productivity.

Beyond enhancing fish health and growth, synbiotic application in aquaculture also provides significant economic and environmental benefits. Improved fish survival and growth rates contribute to higher yields and increased profitability for aquaculture enterprises [28, 32]. Additionally, the use of natural probiotics and prebiotics can reduce dependance on antibiotics and chemical treatments, promoting more sustainable and environmentally friendly aquaculture practices [29, 45, 46]. This transition toward natural and eco-friendly approaches aligns with global efforts to minimize environmental impact and ensure the long-term sustainability of the aquaculture industry.

## 4. Conclusion

This study demonstrates the effectiveness of synbiotic enrichment in *A. franciscana*, particularly through the combination of *E. faecium* and *E. faecalis* with inulin. The enhanced bacterial retention and improved nutritional profile of synbiotic-enriched *Artemia* offer a promising strategy to support early fish development in aquaculture. Further research is needed to assess the long-term benefits and broader applications of synbiotic-enriched live feeds across different aquaculture systems, ensuring their sustainability and effectiveness in improving fish health and growth.

## Figures and Tables

**Table 1:** The growth performance and survival rate of hybrid catfish fed with four experimental diets for 14 days.

Parameters	Treatments			
	T1	T2	T3	Control
Wmg (mg)	44.58 ± 12.99^b^	106.73 ± 53.51^b^	263.14 ± 60.11^a^	126.52 ± 0.01^b^
Lg (mm)	14.14 ± 2.22^a^	11.65 ± 1.44^a^	14.38 ± 0.88^a^	12.32 ± 2.94^a^
SGR (% day^−1^)	5.91 ± 0.64^c^	12.14 ± 1.87^bc^	19.59 ± 1.17^a^	13.36 ± 0.01^b^
S (%)	40.83 ± 13.77^a^	40.00 ± 13.92^a^	57.50 ± 6.61^a^	52.50 ± 7.07^a^
FCR	0.59 ± 0.10^a^	0.35 ± 0.09^b^	0.10 ± 0.01^c^	0.16 ± 0.04^bc^

*Note:* Each value is mean ± SD of three individual observations. Different letters in each row mean significant difference (Duncan's multiple comparison tests, (*p*  < 0.05).

Abbreviations: FCR, feed conversion rate; Lg, length gain; T1, treatment 1; T2, treatment 2; T3, treatment 3; T4, treatment 4; S%, survival rate; SGR, specific growth rate; Wg, weight gain.

## Data Availability

The data used to support the findings of this study are available from the corresponding author upon request.
